# Safety of expanded criteria for endoscopic resection of early gastric cancer in a Western cohort

**DOI:** 10.1186/s12893-018-0414-3

**Published:** 2018-09-25

**Authors:** Rimantas Bausys, Augustinas Bausys, Kazimieras Maneikis, Viktorija Belogorceva, Eugenijus Stratilatovas, Kestutis Strupas

**Affiliations:** 1 Faculty of Medicine, Vilnius University, Ciurlionio str, 21 Vilnius, Lithuania; 2 Department of Abdominal surgery and Oncology, National Cancer Institute, Vilnius, Lithuania; 3 Vilnius University Hospital Santaros Clinics, Center of Abdominal Surgery, Vilnius, Lithuania

**Keywords:** Early gastric cancer, Endoscopic resection, Expanded indications, Safety, West

## Abstract

**Background:**

Endoscopic resection is widely accepted treatment option for early gastric cancer if tumors meet the standard or expanded indications. However, the safety of expanded criteria is still under investigation. Furthermore, discussion, if any additional treatment is necessary for patients who underwent endoscopic resection but exceeded expanded criteria, is rising. This study aimed to evaluate the safety of extended indications for endoscopic resection of early gastric cancer in a Western cohort. Also, we aimed to analyze the lymph node metastasis rate in tumors which exceeds the extended criteria.

**Methods:**

Two hundred eighteen patients who underwent surgery for early gastric cancer at National Cancer Institute, Vilnius, Lithuania between 2005 and 2015 were identified from a prospective database. Lymph node status was examined in 197 patients who met or exceeded extended indications for endoscopic resection.

**Results:**

Lymph node metastasis was detected in 1.7% of cancers who met extended indications and in 30.2% of cancers who exceeded expanded indications. Lymphovascular invasion and deeper tumor invasion is associated with lymph node metastasis in cancers exceeding expanded indications.

**Conclusions:**

Expanded criteria for endoscopic resection of early gastric cancer in Western settings is not entirely safe because these tumors carry the risk of lymph node metastasis.

## Background

Worldwide, the overall incidence of gastric cancer (GC) has steadily declined over the past 50 years, but in some regions (Asia, South America and Eastern Europe) it remained high [1, 2]. Furthermore, the incidence of early gastric cancer (EGC) in these areas is even rising [3]. According to the Japanese classification of gastric cancer, EGC is defined when tumor invasion is confined to the mucosa or submucosa, irrespective of the presence of lymph node metastasis (LNM) [4]. Surgery remains the only potentially curative treatment option for GC, but the extent of surgery for EGC and advanced GC may differ dramatically. Radical gastrectomy with regional lymphadenectomy remains the gold-standard for advanced GC, while endoscopic resection (ER) is sufficient procedure to treat EGC without LNM. According to studies from different regions, the rate of LNM in tumors confined to the mucosa varies between 2.7 and 6.5% and in submucosal tumors between 22.9 and 26.0%. There is a tendency, that rate of LNM in Western countries is higher compared to Asian countries [1]. Since radiological imaging accuracy for LNM detection is insufficient, indications for ER is based on histological tumor characteristics. The absolute indication for ER includes differentiated-type adenocarcinoma without ulcerative findings, of which the depth of invasion is clinically diagnosed as T1a, and the diameter is ≤2 cm [5]. However, only a small part of EGCs fulfill these criteria. The expansion of the standard criteria has been proposed in Japan from clinical observations that too strict indication leads to unnecessary surgery [6, 7]. From the dataset of 5265 patients who underwent gastrectomy for EGC Gotoda et al. identified four additional groups of tumors, which have very low possibility of LNM when they are not accompanied with lymphovascular infiltration [8, 9]. These criteria are described as expanded indications in Japanese Gastric Cancer guideline: 1) differentiated-type mucosal cancer without ulceration and greater than 2 cm in diameter; 2) differentiated-type mucosal cancer with ulceration and up to 3 cm in diameter; 3) undifferentiated-type mucosal cancer without ulceration and up to 2 cm in diameter and 4) differentiated-type submucosal cancer (SM1, < 500 μm from the muscularis mucosae) up to 3 cm in diameter [5] (Table 1).

**Table 1:** The standard and expanded indications for endoscopic resection of early gastric cancer

The absolute indication for endoscopic resection of EGC	The expanded indications for endoscopic resection of EGC
Differentiated-type mucosal adenocarcinoma without ulcerative findings and the diameter is ≤2 cm	1) Differentiated-type mucosal cancer without ulceration and greater than 2 cm in diameter 2) Differentiated-type mucosal cancer with ulceration and up to 3 cm in diameter 3) Undifferentiated-type mucosal cancer without ulceration and up to 2 cm in diameter 4) Differentiated-type submucosal cancer (SM1, < 500 μm from the muscularis mucosae) up to 3 cm in diameter

However, extending the indications for endoscopic EGC treatment remains controversial because the long-term outcomes of these procedures have not been adequately documented [10].

Also, some authors reported LNM in tumors which fulfill extended criteria [3, 10, 11].

*Indications for ER of EGC were established in the Asian population. These findings translation to the Western world may be controversial because two* recently published studies identified non-Asian race as an independent risk factor for LNM [12, 13]. Furthermore, it is not clear if any additional treatment is necessary for patients who underwent ER, but histological examination showed that tumor exceeds expanded criteria.

Therefore, our study aimed to evaluate the safety of extended indications for ER of EGC in a Western population. Also, we analyzed the LNM rate in tumors which exceeds the extended criteria.

## Methods

Regional ethical committee approval was given before study was conducted. Retrospective analysis of prospectively collected GC database was performed. Between January 2005 and December 2015, a total of 1564 patients underwent curative surgery for gastric cancer at the National Cancer Institute, Vilnius, Lithuania. From this cohort, 218 (13.9%) patients underwent open gastrectomy with a D1 or D2 lymph node dissection for early gastric cancer. They were initially enrolled in this study. The clinicopathological characteristics of these patients were reviewed, and 197 patients with tumors who met or exceeded the extended indications for ER were identified and included to further analysis.

### Statistical analysis

All statistical analyses were conducted using the statistical program SPSS 22.0 (SPSS, Chicago, IL, USA). Clinicopathological characteristics were analyzed by the 2-tailed t-test, one-way ANOVA test, Chi-square test or Fisher exact test. Binary logistic regression was performed to identify independent risk factors for lymph node metastasis in the group of patients who exceed the extended indications for endoscopic early gastric cancer treatment. In all statistical analyses, a *p*-value of < 0.05 was considered to be significant.

## Results

Table 2 shows the clinicopathological characteristics of the 218 patients with EGC. 99 (45.4%) patients were diagnosed with intramucosal cancers and 119 (54.6%) with submucosal cancers.

**Table 2:** Clinicopathological characteristics of patients with mucosal and submucosal early gastric cancer

	Mucosal tumor invasion (*n* = 99)	Submucosal tumor invasion (*n* = 119)	*p* value
Age (mean ± SD)	63.5 ± 12.9	67.3 ± 11.6	**0.024**
Gender			
Male	44 (44.4%)	73 (61.3%)	**0.014**
Female	55 (55.6%)	46 (38.7%)	
Histology			
Differentiated	53 (53.5%)	61 (51.3%)	0.786
Undifferentiated	46 (46.5%)	58 (48.7%)	
Lauren classification			
Intestinal	54 (58.7%)	69 (60.5%)	0.902
Mix	8 (8.7%)	11 (9.6%)	
Diffuse	30 (32.6%)	34 (29.8%)	
Lymphanodectomy			
D1	11 (11.1%)	12 (10.1%)	0.828
D2	88 (89.9%)	107 (89.9%)	
No. of retrieved lymph nodes (mean ± SD)	19.4 ± 8.3	20.3 ± 10.7	0.454
Lymph node metastasis			
LNM+	5 (5.1%)	38 (31.9%)	**0.001**
LNM-	94 (94.9%)	81 (68.1%)	
Ulceration			
UL+	30 (30.3%)	48 (40.3%)	0.156
UL-	69 (69.7%)	71 (59.7%)	
Lymphovascular invasion			
LV+	3 (3.0%)	28 (23.5%)	**0.001**
LV-	96 (97.0%)	97 (76.5%)	
Tumor size			
< 2 cm	55 (55.6%)	42 (35.3%)	**0.009**
2–3 cm	25 (25.2%)	40 (33.6%)	
> 3 cm	19 (19.2%)	37 (31.1%)	

All the values in bold shows significance

The rate of lymph node metastasis, the presence of lymphovascular invasion and rate of tumors with greater diameter were significantly higher in submucosal cancer group. 21 patients had tumors which met standard indications for ER and they were excluded from further analysis. 58 patients met and 139 patients exceeded the extended indications for endoscopic EGC treatment. Table 3 shows clinicopathological data of these two groups.

**Table 3:** Clinicopathological characteristics of patients who met and exceeded extended indications for endoscopic early gastric cancer treatment

	Extended indications group (*n* = 58)	Exceeding extended indications group (*n* = 139)	p value
Age (mean ± SD)	65.7 ± 11.3	65.2 ± 12.7	0.438
Gender			
Male	34 (58.6%)	71 (51.1%)	0.352
Female	24 (41.4%)	68 (48.9%)	
Histology			
Differentiated	40 (70.2%)	52 (37.4%)	**0.001**
Undifferentiated	17 (29.8%)	87 (62.6%)	
No. of retrieved lymph nodes (mean ± SD)	20.7 ± 10.8	19.0 ± 7.1	0.212
Tumor invasion			
Mucosal	44 (75.5%)	34 (24.5%)	**0.001**
Submucosal	14 (24.1%)	105 (75.5%)	
Lymph node metastasis			
LNM+	1 (1.7%)	42 (30.2%)	**0.001**
LNM-	57 (98.3%)	97 (69.8%)	
Ulceration			
UL+	8 (13.8%)	70 (50.3%)	**0.001**
UL-	50 (86.2%)	69 (49.7%)	
Lymphovascular invasion			
LV+	0 (0%)	31 (22.3%)	**0.001**
LV-	58 (100%)	108 (77.7%)	
Tumor size			
< 2 cm	34 (58.6%)	41 (29.4%)	**0.001**
2–3 cm	16 (27.6%)	49 (35.3%)	
> 3 cm	8 (13.8%)	49 (35.3%)	

All the values in bold shows significance

Groups were comparable only according to age, retrieved lymph node number, and male: female ratio.

Of 58 cancer who met extended criteria, one (1.7%) had lymph node metastasis in 2 of 22 retrieved lymph nodes. It was not ulcerated, moderately differentiated mucosal cancer with greater than 2 cm diameter (2.2 × 1.8 × 1.5 cm).

LNM was found in 42 (30.2%) of 139 tumors who exceeded the extended criteria. Submucosal tumor invasion (36.2% vs. 11.8%, *p* = 0.009) and presence of lymphovascular invasion (61.3% vs. 21.3%, *p* = 0.001) was revealed as risk factors for LNM at univariate analysis (Table 4).

**Table 4:** Univariate analysis of risk factors for lymph node metastasis in patients who exceed extended indications for endoscopic early gastric cancer treatment

	LNM- (*n* = 97)	LNM+ (*n* = 42)	p value
Age (mean ± SD)	64.6 ± 12.6	66.4 ± 12.8	0.441
Gender			
Male	53 (54.6%)	18 (42.9%)	0.268
Female	44 (45.4%)	24 (57.1%)	
Histology			
Differentiated	39 (40.2%)	13 (31.0%)	0.344
Undifferentiated	58 (59.8%)	29 (69.0%)	
Lauren classification			
Intestinal	50 (51.6%)	18 (42.9%)	0.553
Mix	11 (11.3%)	7 (16.7%)	
Diffuse	36 (37.1%)	17 (40.4%)	
Tumor invasion			
Mucosal	30 (30.9%)	4 (9.5%)	**0.009**
Submucosal	67 (69.1%)	38 (90.5%)	
Ulceration			
UL+	50 (51.6%)	20 (47.6%)	0.670
UL-	47 (48.4%)	22 (52.4%)	
Lymphovascular invasion			
LV+	12 (12.4%)	19 (45.2%)	**0.001**
LV-	85 (87.6%)	23 (54.8%)	
Tumor size			
< 2 cm	30 (30.9%)	11 (26.2%)	0.319
2–3 cm	31 (32.0%)	19 (45.2%)	
> 3 cm	36 (37.1%)	12 (28.6%)	

All the values in bold shows significance

Binary logistic regression confirmed univariate analysis findings and showed submucosal tumor invasion (OR = 5.57, 95% CI: 1.40–22.08, *p* = 0.014) and lymphovascular invasion (OR = 7.13, 95% CI: 2.46–20.64, p = 0.001) as independent prognostic factors for LNM.

## Discussion

EGC treatment with traditional gastrectomy and lymphadenectomy leads to excellent oncological outcomes. Several studies reported 5-year overall survival rate of up to 99% [14, 15]. However, compared to ER, surgery has some disadvantages. It is more invasive treatment method, associated with higher costs and reduced quality of life [16].

Avoidance of unnecessary surgery for appropriately selected EGC patients would lead to treatment improvement. Ideal selection of candidates for ER or surgery would consist of reliable preoperative radiological imaging and identification of LNM before choosing an appropriate surgical method for the individual patient. Unfortunately, available methods are not sufficiently accurate. Currently used endoscopic ultrasonography and computed tomography can reach only 50–87% accuracy [3, 17]. Therefore, the indications for ER is based on LNM risk presumption based on a set of histological tumor characteristics. As mentioned in the introduction section, several reasons exist to consider if expanded indications are entirely safe, especially in the Western population. A study published by Jee et al. [3] confirmed this uncertainty when reported 2.8% LNM rate in a cohort of patients who underwent gastrectomy for ECG which met the extended indications for ER. Alike, data from our present study showed 1.7% LNM rate in the similar cohort.

Furthermore, Jee et al. [3] showed the risk of LNM in three of four expanded criteria, but not in differentiated-type mucosal cancer, without ulceration, greater than 2 cm in diameter. Therefore, authors proposed to consider this indication as safe [3]. In contrast, our study showed that this criterion also carries the risk of LNM. Thus, our result together with previous Jee et al. [3] findings indicates that possibility of LNM exists in every extended criterion.

Two recent studies showed the non-Asian race as a risk factor for LNM in gastric cancer [12, 13]. Our study cohort was very homogenous according to race and ethnicity. All patients were a Caucasian race. Despite, we failed to show a higher rate of LNM in tumors who meet extended criteria compared to the rate reported from similar Asian study [3]. These unexpected findings, together with a fact, that GC incidence in Eastern Europe is significantly higher compared to the rest of Western world, perfectly illustrates heterogenicity of the disease between different regions and different populations. Therefore, multicenter studies with large sample sizes from different racial and ethnical populations are needed to understand the risk of nodal involvement in EGC better. Only new and high-quality evidence will let us establish accurate and reliable clinical practice guidelines for EGC management.

While LNM risk in patients who meets expanded indications for ER is relatively low, patients who exceed these criteria are at high risk. We founded LNM in 30.2% of tumors who exceeded the expanded criteria. Nowadays ER for those tumors is considered as a non-curative treatment. However, some authors discuss that even non-curative ER could lead to satisfactory clinical outcomes. A large multi-center study published by Hatta et al. [18] compared long-term outcomes of patients who underwent either additional radical surgery or only follow-up after non-curative endoscopic resection. Results of the study showed that patients who underwent additional radical surgery had better 3- and 5-year overall survival (OS) and disease-specific survival (DSS) rates. Obviously, it should be declared, that the difference in DSS rates was rather small (99.4% vs. 98.7%) compared to the difference in OS rates (96.7% vs. 84.0%). Also, the rates of recurrence were significantly different, although in both groups they were low - 1.3% and 3.1% in the radical surgery group and the follow-up group, respectively. However, good outcomes in the follow-up group according to DSS and recurrence rates should be treated carefully due to different background characteristics of the study groups. Some major risk factors for LNM (lymphatic invasion or deeper submucosal invasion) were significantly more frequent in the additional radical surgery group [18, 19], and these differences may influence the study results. Furthermore, Suzuki et al. [20] recently published results from the similar study and showed a clear superiority of additional surgery after non-curative ESD compared to follow-up. After propensity score matching analysis, they founded significantly higher rates of 5-year DSS rate (99.0% vs 96.8%) and 5-year OS (91.0% vs. 75.5%) in the additional surgery group [19]. Results of those two studies and a high rate of LNM revealed in our study indicate, that EGC which exceeds expanded criteria for ER should be treated with gastrectomy and appropriate lymphadenectomy.

Some limitations of the present study should be mentioned as well. First, 5 (8.6%) of 58 patients with EGC that met expanded indications for ER underwent D1 lymphadenectomy. Because of limited lymphadenectomy, the risk of LNM in this group could be underestimated. Second, our study sample size was small compared to reports from Asian countries. Only 58 patients were in a group of tumors who met extended criteria for ER. However, lack of reports from Western countries increases the scientific value of our paper. Furthermore, despite the small sample size we managed to reach our study goal and showed the risk of LNM in tumors who meet expanded indications for ER.

## Conclusion

Implementation of expanded criteria for endoscopic resection of EGC in a Western setting is not entirely safe because cancers who meet these indications carry the risk of LNM.

EGC who exceeds expanded indications has a high risk of LNM, therefore gastrectomy with lymphadenectomy should remain a standard treatment option.
